# The short (and messy) life of the protagon theory of anaesthesia

**DOI:** 10.1007/s40656-026-00735-4

**Published:** 2026-05-01

**Authors:** Matthew Perkins-McVey

**Affiliations:** Technion - Israel Institute of Technology, Haifa, Israel

**Keywords:** Anesthesia, Ludimar Hermann, Entity realism, Historical ontology, Pharmacology

## Abstract

GABA_A_ and protein hypotheses aside, the lipid theory remains the most influential account of anaesthetic effects in modern pharmacology. An apparent aspect of the lipoidal model’s continued dominance is its long historical pedigree, frequently aligned with a process of steady refinement from Bibra and Harless to Hermann, Pohl, and finally Meyer/Overton. This paper problematizes this continuum by placing the genesis of a short-lived experimental entity—protagon—at the centre of the transition from the fat-solvation theory to the modern lipoidal theory of anaesthesia, which broadly identifies fat as a central mediating substance. A ‘historical ontology’ of protagon and an outline of the 19th century history of brain composition in relation to the early history of anaesthetic pharmacology, it treats the initial appearance of fat-oriented theories of anaesthesia as emergent of the focus on cerebral fat endemic to early 19th century organic principle analysis.

## Introduction

Prior to the advent of general anaesthesia in the nineteenth century, the trauma and shock of surgery, even with the added benefit of analgesics, posed an enduring barrier to the physical and psychological well-being of all who came under the surgeon’s amphismela, lancet, or saw. In an 1824 publication in *The Lancet,* an anonymous author recalled the experiences of patients for whom, “feverishly heated, and frequently very much exhausted by his previous sufferings,” “every additional moment […] becomes to him an hour, and every additional moment that he continues under the torture of the different instruments, diminishes the chance of success” (Anonymous, 1824, pp. 91–92). Beyond the practical difficulty faced by those operating on patients in such a state, the experience of the surgery itself posed immediate dangers. Worse still than the “cries,” “screaming,” and “agony,” every harrowing moment endured in waking surgery was coloured by the “danger of [their] life” (Anonymous, 1824, p. 92). Operators often strove to complete the surgery as quickly as possible, not merely in order to stave off death by hemorrhaging, but out of concern that the shock itself would comprise the patient (Booser, 2021).

With these images at hand, it is easy to envision in what sense the advent of general anaesthesia was transformative, even revolutionary. Nor was it merely a matter of their tremendous impact on medical practice. They broadened the horizon of human experience. As many remarked at the time, these novel intoxicants elicited radically new kinds of mind states, similar to and yet different from narcosis: what we now think of as anaesthesia (Behrend, 1864; Bibra & Harless, 1847; Glover, 1842; Perkins-McVey, 2024).^1^ Ether had been used as an anaesthetic in conjunction with opium by English physicians as early as 1840, but it was not until Crawford Williamson Long’s (1815–1878) Boston demonstration in 1842 that ether would be used as a general anaesthetic, followed by William T. G. Morton (1819–1868) who, in 1846, became the first to publicly demonstrate the use of ether for surgery (Anaya-Prado & Schadegg-Peña, 2015; Boott, 1847; Grattan, 1840). In 1847, James Robinson completed the first surgery under ether anaesthesia outside of the United States, extracting the molar of a “Miss Lonsdale” in the study of Francis Boott’s London home (Boott, 1847, p. 8). Chloroform, too, entered medical practice on an almost identical timeline (Dunn, 2002; Perkins-McVey, 2024; Rae & Wildsmith, 1997). With its rapid spread in the practical art of medicine, it is no wonder that interest in the underlying nature of anaesthesia almost immediately captured the imagination of those engaged with adjunctive study of bodily chemistry.

It would be no later than 1847—the very year that ether was used as a general anaesthetic in a surgical setting—that Ernst von Bibra and Emil Harless published *Die Wirkung des Schwefeläthers in chemischer und physiologischer Beziehung*, outlining the physio-chemical function of ether anaesthesia. Bibra and Harless’s early study centred on the interaction between cerebral fats and ether in the blood, a basic model which would later be expounded through subsequent fat-based theories of anaesthetic function. The lipoidal theory of anaesthesis is a theory whose organizational significance holds cache to this very today, and it is typical to draw a line of more or less continuous development from Bibra and Harless, to Ludimar Hermann, Pohl, and Meyer/Overton, the last of which is generally aligned with the advent of the modern theory (Hintzenstern et al., 2002). But this continuum glosses over the underlying conceptual dissimilitude found throughout the works of these disparate theorists and, in doing so, loses sight of the nature of their points of congruence. How did brain fat come to be associated with a radically new kind of mind state, so-called anaesthesia, in the first place and what does it have to do with the differences between the conclusions advanced by Bibra and Harless or Meyer/Overton? Answers to both of these questions can be found in the work of Ludimar Hermann, whose controversial decision to ascribe the effects of anaesthetic vapours to a novel, if short-lived, bodily entity—Liebreich’s “protagon”—may have shaped the course of the lipoidal theory as we now know it.

Little discussed outside of occasional histories of anaesthesia written by physicians and generally subordinated to the broader development of the lipoidal theory of anaesthesia, protagon, and especially protagon theory, is a space of historical inquiry in need of greater explication (Hintzenstern et al., 2002; Perouansky, 2012; Sourkes, 1995). This investigation endeavours to bring the long-forgotten protagon to the fore in the history of anaesthesia. At once a historical ontology of protagon, a history of anaesthetic effect, and a case study in the dynamic interplay between experimental entities and theories, this investigation places the genesis of protagon, a now-forgotten bodily entity, at the centre of the radical shift from the fat-solvation theory of anaesthesis to that of cerebral fat as a mediating substance. Centring on one cerebral fat among many in its passing from chemical to physiological, medical, and pharmacological inquiry, it explores how the short and messy ontology, dissemination, and disappearance of a single entity—protagon—helped mediate the relationship between cerebral fat and anaesthesia, a relationship that informs the lipoidal theory of anaesthesia to this day.^2^ Protagon will assumed the role of the *adamic fat,* a concept and entity which was capable of marshalling the evolutionary metaphors which foregrounded scientific discourse in the mid-nineteenth century in accordance with the received research program of cerebral fat, the conceptual and methodological focus of early-nineteenth century organic principle analysis. By the time the once-tangible protagon had passed out of being, it had already fundamentally altered the course of what would come to be the lipoidal theory of anaesthesia. With the stakes marked, let us turn to the natural starting point of such a study and briefly consider the fatty brain as the focus of the research program of organic principle analysis.

## Proximate principle analysis: the fatty brain

Well before Bibra and Harless could even think to scrutinize the fatty content of the brain, French and German chemists of the late-eighteenth century were mired in a series of methodologically arduous investigations into the chemical constitution of organic materials. Every discernible part of the organism was to be broken down and classified according to its constitutive principles, its basic, most reducible components. A flower was no longer an entity unto itself, or even a vegetative stalk with petals and a pistil, but a sprawling *milieu* of loosely connected components, each formed by distinct compounds. Though elementary analysis and the search for simple principles often foregrounded classificatory efforts in accordance with the Lavoisierian program of analytical chemistry, the investigatory methods and approach to classification in proximate principle analysis varied widely, even within associated groups (Klein & Lefèvre, 2007; Lesch, 1981).^3^

France undoubtedly led the pioneering research program of organic proximate principle analysis. Classifying this swelling compendium was not merely compelling on a theoretical level, but bore immediate significance in pharmacy and medicine. Empire, exploration, colonization, and increasingly global trade had constantly added to the lists of known plant and animal entities (Lesch, 1981). At the same time, pharmaceutical chemistry was then still a practical science in the late-18th/early-nineteenth century Germany and Britain (Rocke, 2023). France was unique for the extent to which chemistry and pharmacy had become institutionalized, owing in large part to the legacy of Lavoisierian chemistry. It thus comes as little surprise that it was Antoine-Francois Fourcroy, a former member of the pre-revolutionary Paris Sociéte de Pharmacie and a founding figure of the post-revolutionary Paris Ecole Supérieure de Pharmacie, who first realized organic principle analysis as an organized body of research. Fourcroy simultaneously ordered plant principles according to their appearance in the life cycle and their chemical characteristics, strictly acknowledging the validity of principles which could be extracted without change (Fourcroy, 1800; Lesch, 1981). While this method of categorization might at first appear arbitrary, it accorded with the practical and theoretical concerns of pharmaceutical chemistry, the institution’s titular focus. In contrast, the Scottish chemist Thomas Thomson sorted plant components according to their solubility in ether, water, or alcohol, consistent with his interest in the chemistry of inorganic bodies in general and mineral chemistry in particular (Thomson, 1809).

Fourcroy himself realized that his classification method would rapidly be made obsolete and it was many of Fourcroy’s own students who set upon developing an approach to categorizing organic principles that appeared more systematic (Klein & Lefèvre, 2007; Lesch, 1981). Louis Jacques Thénard, a student of Fourcroy’s, turned to an elemental basis for classification, divided proximate plant principles composed of nothing more than carbon, oxygen, and hydrogen into substances with a relative proportion of oxygen to hydrogen that was greater than water; those in which they were in equal proportion; and those in which there was a lesser proportion of oxygen to hydrogen (Lesch, 1981; Thénard, 1817).

Organic principle analysis soon emerged as an object of research outside of France, particularly among those engaged with the developing field of pharmaceutical chemistry. Friedrich Stromeyer, educated at Fourcroy’s school in Paris, and Sigismund Friedrich Hermbstädt, an aspiring pharmaceutical chemist apprenticed at Trommsdorff’s pharmacy, were the foremost representatives of this approach in the Germanies. Hermbstädt published various methods for the isolation of proximate plant principles and supported boundaries between naturally occurring plant principles and other materials, although his classificatory approach maintained a traditional focus on external physical and chemical attributes (Klein & Lefèvre, 2007). Stromeyer was comparatively radical in his determination to order substances according to their apparent chemical constitution, freely placing various ethers and alcohols alongside plant-gums and vegetable acids under the column reserved for oxygenated compound bases (Klein & Lefèvre, 2007; Stromeyer, 1808).

Largely centred as they were around pharmaceutical chemistry, early organic principle analysis was characterized by a decided lack of classificatory consensus. For all of the classificatory chaos that characterized late-18th and early-nineteenth century organic analysis, these disparate efforts nonetheless shared in a basic epistemological commitment. Emergent of broader seventeenth and eighteenth century changes in the understanding of nature, which now saw in everything something to be measured, weighed, and classified, organic analysis was united in its understanding of where to look, what looking meant, and what was important to look for. As for what they found, the absence of a generic nomenclature, common approach, or even internal classificatory consistency was not an apparent issue (Klein & Lefèvre, 2007; Lesch, 1981; Löw, 1977). Much as how the seventeenth century advent of probability saw the application of probabilistic thinking in the determination of considerations bereft of statistical significance, what mattered was not how they classified their findings (that came later), but that they were classified (Hacking, 2006). Anything less was only part of the picture.

Of course, the research paradigm of elemental analysis did not solely figure on plant principles. Though the proximate plant principles captured the bulk of the research program’s initial focus, the conceptual and methodological bases of elemental analysis further extended into animal principles and inorganic chemistry. To expand on a ready example, within the aforementioned class of oxygenated compound bases, Stromeyer further placed the proximate principles of animals, such as *Eyweißstoff,* gelatin, and urea (Klein & Lefèvre, 2007; Stromeyer, 1808). Elemental analysis was predicated on an epistemic commitment which placed the *locus* of the real in the constitutive elements of organized beings.

Research into animal principles quickly zeroed in on the complex subject of nervous tissue, extending a methodological program rooted in pharmaceutical chemistry into the chemistry of the body. In 1811, Louis Nicholas Vauquelin, assistant to Fourcroy in Paris, published “Analyse de la matière cérébrale de l'homme et de quelques animaux,” outlining the process by which Vauquelin extracted a fatty substance from brain tissue. Vauquelin’s extract was simply referred to as “matière blanche,” which was insoluble in alcohol or water (Vauquelin, 1811, p. 19). In organizing components of brain tissue relative to their solubility in alcohol or water, Vauquelin’s classificatory method was consistent with those of Fourcroy and French pharmaceutical chemistry. What mattered was that Vauquelin had successfully extracted some kind of novel organic principle from brain tissue, a new kind of bodily entity. Vauquelin’s results were soon repeated by Johann John in 1814 through an extensive study of the brains of poultry, crayfish, calves, and deer (John, 1814).^4^

Modestly begun as a cadet branch of the broader interest in organic principle analyse among pharmaceutical chemists, Vauquelin’s matière blanche rapidly emerged as an object of interest in the separate fields of physiology and the related field of animal chemistry. In 1826, the chemist Leopold Gmelin, a former assistant of Stromeyer, and the physiologist Friedrich Tiedemann sought to understand the metabolic processes by which food was incorporated into the living organism (Grote, 2020; Stumm, 2012). They eventually turned to the analysis of brain matter, yielding a pulverulent fat and crystalline isolate, which they termed Cholesterin, and which they had previously discovered in animal bile (Gmelin & Tiedemann, 1826; Stumm, 2012).^5^ Shortly after, Otto Bernhard Kühn, another student of Stromeyer and a lecturer in theoretical chemistry in Leipzig, attempted an extraction from matière blanche using ether, naming the extract cerebrin and referring to the insoluble fat as myelokon (Kühn, 1828). At first an exploratory entailment of what was largely pharmaceutical chemistry, matière blanche had taken on a life of its own, now a subject of decisive in animal chemistry.

Investigative fervour surrounding particularly the fatty components of nervous tissue only increased in the 1830s and 1840. In 1834, J. P. Couërbe, an animal chemist, presented the findings of his dissertation to the Académie des Sciences under the title “Du cerveau considéré sous le point de vue chimique et physiologique” (Couërbe, 1834). While it cannot be denied that such considerations weighed on the thoughts of preceding chemists, Couërbe was among the first to explicitly align the study of cerebral fatty tissue with "the question of life, of intelligence" (Couërbe, 1834, p. 2).^6^ Fatty brain tissue was no mere theoretical interest of analytic chemistry. It was located in the heartland of grand and enduring inquiries of spiritual, philosophical, and scientific importance. The central object of animal chemistry should not be, by Couërbe’s reckoning, the chemistry of muscles or tears, but these greater questions, the answers to which could be found nowhere but the nervous system (Couërbe, 1834). Dissatisfied with the present state of animal chemistry, much of which Couërbe deemed “futile, leading to no satisfactory physiological explanation,” Couërbe’s research enrolled an array of methodological approaches yet unseen in the study of nervous tissue (Couërbe, 1834, p. 1).^7^ The objective of Couërbe’s project was not only to extract proximate principles from brain pulp, examine them microscopically, and study their chemical composition, but to identify a physiological difference in the brain matter of asylum patients (Couërbe, 1834).^8^ This study further represented the first quantitative elementary analysis on brain matter, making use of Liebig recently developed combustion analysis technique (Couërbe, 1834). Couërbe described a white, fatty substance “cérébrote” (which he took to be Vauquelin’s isolate), in addition to a powdery yellow grease called “stéaroconote” (possibly the pulverated fat mentioned early), a stretchy fat dubbed “céphalote,” and a reddish oil named “éléencéphol” (Couërbe, 1834).

Further investigations were undertaken by Vauquelin, as well as the chemists Jöns Jacob Berzelius and Edmond Frémy, in the 30 s and early 40 s, with Frémy quite harshly contradicting Couërbe’s findings and declaring cérébrote to be an acid, which he subsequently called *acide cerebrique*, or Cerebrinsäure (Frémy, 1841; Liebig, 1842, p. 109)^9^ But the zenith of early nineteenth century inquiries into nervous fats was arguably those of Théodore Nicolas Gobley, protégé and later son-in-law to the famous pharmaceutical chemist Pierre Jean Robiquet. From 1843–1874, Gobley published elementary analyses on a successive diversity of animal tissues, writing on egg yolk in 1847, carp eggs and roe in 1850, as well as the brain matter of various vertebrates in 1850, 1852, and 1856. Extending his 1845 discovery of the lipoidal contents of egg yolks, Gobley proposed in 1847 that the lipid contents of yolk consisted of both Vauquelin’s *matière blanche* and a new, stable—but yet unrealized—*matière phosphorée* (Gobley, 1845, 1847a). That same year Gobley extracted his *matière phosphorée* from brain matter, and by 1850 had dubbed it lecithin, after the Greek word for egg yolk (Gobley, 1847b, 1850; Zeisel, 2012).

This dizzying chronicling of names serves a purpose. Fat-like materials in the brain—their constitution, nature, and significance—had become the kind of thing to figure out. This is made as apparent by the rapidity with which we witness claims to the ontology of novel entities as by the competitiveness that diffused each publication. A single mention is typically not enough to constitute an ontology. What we find pulled from the pages of each new publications are ontological claims, ones then borne out in the interplay between the new kind, experimental phenomena, and communities of knowledge. Such spheres of negotiation are predictably rife with conflict as ontological claims converge on increasingly similar points. In this regard, the proximate principle analysis of cerebral fats was no different. It might have been said, for example, that the matière blanche of Vauquelin was the same substance that Couërbe named cérébrote, or that Kühn called myekolon. There were scattered efforts to accommodate, if not unify, this diversity of named cerebral entities, many of which were suspected to be identical. For example, Kraus’s, (1844) *Kritisch-etymologisches medizinisches Lexikon* defined "cerebrina, cerebrine, cerebrinum, die Cerebrine, das Cerebin" as well as "cerebrotum, le cèrèbrote, das Cerebrot" as an essential fatty substance found in brain matter (Kraus, 1844, pp. 187–189). In short, the ontologies of some entities were, for one reason or another, more suspect than others, reflecting a vague awareness that this fertile sub-field of research might have become too productive.

And yet, investigative efforts renewed themselves again and again. The public rivalries and disagreements between individual researchers which surrounded the identity of fatty brain principles, such as the heated disputes seen between Couërbe and Frémy or Kühn and Gmelin, only further illustrated the perceptions of timeliness and necessity which spurred these enterprises. A parallel might be drawn with the famously raucous debate between Louis Pasteur and Robert Koch. What began with pharmaceutical chemistry’s interest in organic principles had become one of the most heated research programs in animal chemistry. At stake was much more than eminence on a current problem in animal chemistry. It was a matter of priority in what may yet provide answers to the question of life itself.

It would not be long before the widespread interest in the physio-chemical significance of cerebral fat had a direct influence on physiology and medicine. When ether seeped into German medical awareness, a mere two years before Bibra and Harless’s study, fatty masses in the brain were a rational place to look, particularly for investigators who found themselves outside of developing centres of experimental physiology. The question, then, is not why did Bibra and Harless turn to fatty matter in the first place. That had already been established through the expansive proliferation of experimental studies on the fatty contents of the cerebrum and the entailing promotion of the topic as a subject of significance. Situated in their broader scientific context, it was simply the natural place to look. For Bibra and Harless, the question was what fat-like substances *became* in the process of their theoretical entanglement with an emerging theory of narcosis.

## Bibra and Harless’s fat solving theory

Ernst Freiherr von Bibra and Emil Harless do not at first present as the most likely popularizers of a cerebral fat based theory of anaesthetic effect, or, as they would say, ether narcosis. Harless was a physiologist, but only barely, having completed his doctorate the year prior. Bibra, on the other hand, was concerned with all manner of topics from zoology and geography to physiology and chemistry, representing every bit the dying breed of the aristocratic polymath and Romantic adventurer.^10^ Some will be familiar with the story of how, in a series of experiments on animals, Bibra and Harless found that etherization was accompanied by a decrease in the fatty composition of the brain and spinal cord, alongside a simultaneous increase in the amount of fat found in the liver (Bibra & Harless, 1847; Hintzenstern et al., 2002) Devising a respirator and face mask, Bibra and Harless first etherized themselves, detailing its subjective human effects, before elaborating on ether’s physiological effects through a series of animal experiments involving rabbits, cats, rats, pigeons, sparrows, several frogs, and one large salamander (Bibra & Harless, 1847).^11^ Where possible, they measured pulse rate, changes in the iris, and urine output, followed in the majority of cases by dissection to observe the influence of ether on the brain and other organs (Bibra & Harless, 1847). The fatty contents of the brain appeared to shrink, or even dissolve, when ether was introduced, possibly owing to ether’s exceptional effectiveness as a solvent (Bibra & Harless, 1847). Further observing alterations in the chemical composition of urine samples taken during and immediately following the abatement of ether narcosis, Bibra and Harless noted a simultaneous change in metabolism, which they associated with the mobilization of brain fats (Bibra & Harless, 1847; Hintzenstern et al., 2002).^12^ It was quite apparent that, on the chemical level, the phenomenal experience of ether narcosis was the product of some alteration in the fatty contents of nervous tissue and the resultant stimulation of the metabolic process.

For Bibra—and perhaps Harless as well, this realization of fatty material in the brain as *the* critical physiological actor behind the phenomenon of ether narcosis was remarkably influential. Fat soon emerged as a central bodily principle, involved in the governance of many aspects of animal life. With cerebral fats already a well-established subject of scientific inquiry and the findings of his ether studies in hand, Bibra soon turned to the subject of the brain for himself. In 1854, Bibra published *Vergleichende Untersuchungen über das Gehirn des Menschen und der Wirbelthiere,* a comparative analysis of the relative concentrations of fats, waters, and fixed components in the nervous tissues of humans and animals (Bibra, 1854). The text centred on Bibra’s own histological and anatomical studies of various human cadavers, coupled by his study of an uncommonly diverse array of living and dead birds, rodents, amphibians, sheep, dogs, goats, horses, and other livestock (Bibra, 1854). Far from simply integrating the findings of foregoing chemical studies, Bibra conducted his own chemical analysis of the phosphorus content of cerebral fat (Bibra, 1854, p. 97).^13^ Though technically devoted to the broader brain, this focus on the centrality of cerebral fats pervades Bibra’s book. No later than the first sentence of the very first page, Bibra asserts that, although “the physiological importance of the brain does not depend on fat alone, *it is largely determined by it*” (Bibra, 1854, p. 1).^14^ Not only was “fat an integral component of the brain,” it “is designated to play a completely different role in the brain that in most other organs of the animal body” (Bibra, 1854, p. 1).^15^ Bibra would further offer that the higher an animal the greater the fatty contents of their brain, suspecting that higher animals with lighter brains made up the difference through a greater proportion of fat (Bibra, 1855, p. 82).

It is clear that chemical analysis of fatty substances in the brain influenced Bibra and Harless’s study of the relationship between ether and fat, and even further cemented the physiological significance of cerebral fats. This does not explain why cerebral fat came to be associated with anaesthetizing drugs, while other intoxicating substances generally did not. To gain a full appreciation of the role Bibra accorded to fat in nervous function, one needs to consider one of Bibra’s most famous works: *Die Narkotischen Genussmittel und der Mensch* (1855). Here, Bibra feels free to extend his fat-based theory of ether anaesthesia, straining the reach of his theory far beyond experimental observations. Chloroform and alcohol were natural bedfellows of the theory, as “chloroform behaves very similarly to [ether] and bringing the whole range of alcoholic drinks into the area of our theory was an obvious thought” (Bibra, 1855, p. 82).^16^ But Bibra lets fly the arrow of speculation far afield the modest inclusion of further commonplace solvents. Bibra admits to “know very well that a number of other substances act on the nerves which do not possess the fat-dissolving properties of ether, alcohol and etheric substances;” yet, “this does not, however, exclude the possibility that these [substances] have an effect in an implied manner” (Bibra, 1855, p. 83).^17^

It would be easier to concede to Bibra’s rhetoric, and regard such remarks as being merely *ad ignorantium,* were he not so eager to offer possible points of connection between the fat-theory and the observed effects of other intoxicants. Concerning the effects of coffee and tea, Bibra concedes that these substances do not appear to have any effect on brain fat. However, coffee and tea did appear to slow the metabolism, as shown by Böcker’s research into tea’s effect on carbon dioxide and urine output (Bibra, 1855). As the effects of coffee and tea are undeniably less acute that those of ether, Bibra left open the possibility that some aspect of the tea or coffee brings about a barely detectable alteration in the brain fat (Bibra, 1855, p. 83).^18^ The consumption of opium is likewise associated with a pronounced metabolic shift, further noting the exceptional solubility of the various opium alkaloids in animal fat (Bibra, 1855).^19^ Opium smoking, in particular, is alleged to waste the fat of those who consume it, transforming solid body fat into “an oily, fluid mass” before it disappears altogether, an observation which Bibra extracted from the Singapore travel reports of the physician Robert Little (Bibra, 1855, p. 211; Little, 1848).^20^ With no meaningful alternatives presented, Bibra appears to suggest, with varying degrees of directness, that the fat-theory might account for the entire range of effects engendered by intoxicating substances, that the discoveries made alongside Harless ultimately cast a brighter light upon the shadow-world of pharmacological activity than previously suggested.^21^

Though Bibra and Harless were methodologically concerned with explaining the phenomenon of ether narcosis on a physio-chemical level, their broad theoretical interest in the topic drew on ether’s implications for Romantic philosophy. German Romantic thought represents a broad and, at times, loosely connected network of approaches, concepts, and perspectives. Nevertheless, a genre-defining feature of early nineteenth century German Romantic thought was its focus on reflexivity or the dialectical (Bortoft, 1996, 2012; Perkins-McVey, 2026). Arguably almost everything, and certainly living consciousness, was not to be understood as a thing in itself, but emergent of some dialectical process between inner and outer, between self and world (Bortoft, 1996, 2012; Perkins-McVey, 2026).^22^ Ether, on the other hand, appeared to physiologically intercede in the midst of this dialectical process, somehow blocking the interplay between spirit and world that gives rise to lived consciousness.^23^ “Self-consciousness in itself is an absurdity,” they reminded readers, “it cannot be sustained without those two interconnected objects of our imagination, one of which is outside us and the other within ourselves” (Bibra & Harless, 1847, p. 81).^24^ It is only *vis-à-vis* the imposition found in interacting with an external world that self-consciousness becomes possible in the first place.^25^ This experience of an embodied, spatially circumscribed, and determinate selfhood is “physiologically mediated through the activity of the sensory nerves on the one hand, and the motor nerves on the other hand, which can be excited by the central organ according to their mood at any given time” (Bibra & Harless, 1847, p. 82).^26^ The sensory-nervous apparatus, thus, fords an otherwise impassable torrent, occasioning the dialectical engagement between *Geist* and world.

In ether narcosis, this dialectic interplay between self and world is all but washed away. Rapidly muting the senses, ether abolishes self-awareness, suppresses consciousness, and—finally—halts muscle activity (Bibra & Harless, 1847; Hintzenstern et al., 2002). At the point where ether abolishes self-consciousness, “the activity of the sense organs disappears,” “willpower no longer activates the motor nerves, and the soul is left restlessly busied with the creation of affectless ideas and images” (Bibra & Harless, 1847, p. 82).^27^ Delivered from the sensory world that is the reflex basis of self-conciousness, the subject’s mind floats freely through series of dream-like images, the likes of which are scarcely memorable after ether’s effects have abated (Bibra & Harless, 1847, p. 83). It was apparent that ether produced its effects through some alteration in the nervous tissues presiding over the perceptional interface between self and world, affecting not merely the activity of the motor nerves but their basic sensibility (Bibra & Harless, 1847; Hintzenstern et al., 2002). Ether narcosis itself thus illustrated the Romantic conception of self-consciousness as emergent of the dialectical reflexivity between inner and outer, further mirrored by the sensory apparatus’s central role as both the soul’s physical counterpart and condition of possibility (Bibra & Harless, 1847).

Such properties were, undoubtedly, remarkable to Bibra and Harless.^28^ Echoing Robert Glover’s entitling of chloroform and other halogenous compounds as a “new class of poisons” in 1842, Bibra and Harless recognized ether narcosis to be a “a new ‘category’ of mental states,” a radical departure from the encounters then available in the *materia medica* (Bibra & Harless, 1847, p. 83; Glover, 1842; Perkins-McVey, 2024).^29^ Though Bibra and Harless’s theoretical conclusions were far from universally accepted, their work garnered recognition from a diversity of audiences, including the Paris Academy of Science, and even their detractors recognized that it was “the richest text” on the subject yet written (Klencke, 1848; Lösch, 1848, p. 4; Martin, 1847).^30^

## Protagon: the adamic fat concept

The work of Bibra and Harless thus more generally falls among the flurry of investigations centred on cerebral fats. At the same time, their study represented a bold departure from the limited purview accorded to cerebral fat in the field of organic principal analysis. Bibra and Harless’s experiments drew cerebral fats from their initial context as a subject of chemical inquiry into the new contexts of medicine and physiology. Once there, they further mobilized cerebral fats, both as literal bodily entities and conceptual actors, in the pursuit of new kinds of questions, marking a distinct effort to mobilize these insights around the physiological phenomenon of ether narcosis. Investigations into the constitution of animal tissues continued, with Adolf Strecker examining bilious lecithin at the University of Tübingen in 1862. Strecker observed that, when heated to the point of boiling, bilious lecithin gave way to a novel nitrogenic chemical, which he named “choline” in reference to its cholic origins (Zeisel, 2012). It was a mere three years later that Oscar Liebreich, likewise an assistant in Tübingen's Schlosslaboratorium, published the findings of his studies on the chemical constitution of the human brain (Liebreich, 1865). Liebreich made the remarkable declaration that the entire host of Lavoisierian forbears had erred.^31^ “Those bodies that are named cerebrin, Cerebric acid, lecithin, etc. and the fats known as phosphoric fats *do not primarily exist in the brain*” (Liebreich, 1865, p. 29).^32^ All of these proposed principles of nervous tissue were, in fact, either the products of decomposition in the dead brain or were simply not true independent chemical entities (Liebreich, 1865).

In support of this sweeping claim, Liebreich accurately or inaccurately claimed that none of the preceding researchers extracted samples from freshly killed animals (Liebreich, 1865). Decomposition began almost instantly. Waiting minutes, hours, or days before examining a sample would produce nothing but decomposition products (Liebreich, 1865). Plucked from the brain paste of a freshly killed animal, Liebreich instead claimed to have identified the singular fatty substance from which all other brain isolates were derived, the originating substance of all neural products, a massive molecule with the formula C_116_H_241_N_4_O_22_P (Liebreich, 1865, p. 32). “Wherever earlier authors refer to glycerol phosphoric acid, cerebrin, etc., and the so-called 'enigmatic phosphorus-containing fats',” this substance is at play, “for example in the yolk of eggs, ovaries, sperm, etc.” and “from this [substance] comes the microscopic form observed by Virchow, to which he gives the name myelin” (Liebreich, 1865, p. 39).^33^ Liebreich dubbed this substance “Protagon (from *πρωταγον*)” (Liebreich, 1865, p. 30).^34^ Liebreich’s, 1865 paper aspired to nothing less than an ontology of *the* brain molecule.

Following these statements with a series of methodological critiques of these earlier investigations, Liebreich had, in one fell swoop, folded decades of proximate principle analyses on nervous tissue into a single entity.^35^ Protagon was not unique among the proposed cerebral fats, all of which might variously be described as scientific objects, as epistemic things, emergent of discrete assemblies of experimental systems (Rheinberger, 1997). After all, Liebreich himself undermined the ontological claims of further cerebral fats on methodological grounds. Liebreich’s experimental system, containing within it all of the specific experimental refutations levelled against half a century of research, saw the genesis of (yet another) thing that had previously not existed. But Liebreich’s protagon was distinct among the proposed cerebral fats in its categorical and rhetorical scope. At the top of it all, Liebreich proposed, was fatty protagon, the *adamic proto-fat*, “the mother-substance-of-all,” the originary progenitor of all the particular chemical bodies enlisted in brain activity (Liebreich, 1865).^36^ The appeal of this streamlined approach is reflected in its ready uptake.^37^ A year later, the contents of leading journals were replete with studies which, at least outwardly, assume the veracity of Liebreich’s claims. Of particular significance was protagon’s almost immediate reception in physiology, where protagon would soon feature in a novel theory of anaesthetic action.

## Protagon in the blood: Ludimar Hermann’s theory of anaesthesia

One of protagon’s earliest champions was the physiologist, later toxicologist, Ludimar Hermann, then still counted among Emil du Bois-Reymond’s best pupils at the University of Berlin (Finkelstein, 2006).^38^ While others were slow to directly engage protagon with a theoretical framework, Hermann would almost immediately put protagon to work, specifically in explaining the effects of various intoxicating substances. Published a year after Liebreich’s inaugural protagon paper, Hermann’s “Ueber die Wirkungsweise einer Gruppe von Giften” represents the first protagon-based theory of anaesthetic action (Hermann, 1866b). Far more than a simple substitution of protagon for an undefined group of cerebral fats, the advent of the fleeting protagon concept saw the emergence of a radical shift in the basic understanding of pharmacological action. The protagon theory would draw on Darwinian metaphors and the historical givenness of fat theory in translating cerebral fats from passively soluble substances into an active mediating substance, the causative agent behind the powerful mind-states engendered by anaesthetic substances.

The immediate appeal of the protagon theory for Hermann likely stemmed from the concept’s organizational potential for Hermann’s prior and ongoing research into the physiological absorption of “Gase der Muskeln” (gases of the muscles) (Hermann, 1865). The electrophysiological function of the body, and particularly the muscles, constituted the main research focus of Hermann’s mentor and teacher, du Bois-Reymond (du Bois-Reymond, 1848; Finkelstein, 2006, 2014). Du Bois-Reymond published widely on the physio-chemical nature of the muscles, as well as the methodology of blood analysis, and Hermann, as a junior scientist under du Bois-Reymond’s supervision, was still actively expanding on his teacher’s work (du Bois-Reymond, 1848, 1854, 1863; Finkelstein, 2014). The role of absorbed gases in the blood and muscle was in this sense an appropriate line of inquiry for the young Hermann, all the more because the topic had received relatively poor attention after the foundational work of Humphry Davy and Johannes Müller in the earlier half of the century.

Thus, in the year preceding Liebreich’s protagon paper, Hermann’s “Ueber die physiologischen Wirkungen des Stickstoffoxydulgases” mobilized a physicalist inquiry against the “merkwürdige” (peculiar) observation of Humphry Davy—later supported by the famous physiologist Johannes Müller—that nitrous oxide could temporarily sustain life, though it would eventually result in death (Hermann, 1864, p. 521).^39^ Hermann disputed these observations by reframing the Davyian experiment as a question of the respirability of nitrogen-bound oxygen (Hermann, 1864). The absorption coefficient of nitrogen-bound oxygen in blood was proportionally equivalent to the water content of blood—merely absorbable, and thus incapable of sustaining life. That feelings of dyspnea were not immediately experienced following the inhalation of nitrous oxide was simply a product of nitrous oxide’s anaesthetising effects (Hermann, 1864).

Hermann’s inquiry into the respirability of nitrogenic oxygen was subsequently followed by “Ueber die Wirkungen des Stickstoffoxydgases auf das Blut,” turning the investigation toward some of the ambiguities surrounding nitrous oxide’s absorption in the blood. With some clever glasswork and the help of a baryte solution to mitigate the formation of hyponitrous acid, Hermann was able to qualitatively demonstrate that N_2_O turned oxygenated blood a “beautiful bright crimson colour,” similar and yet distinct from the purple hue of deoxygenated blood (Hermann, 1865, p. 472).^40^ With continued experiments, Hermann explained this phenomenon as the result of hemoglobin’s readiness to bind with N_2_O, and that the blood’s affinity for N_2_O was in fact so strong that it readily displaced oxygen and even competed with CO_2_ (Hermann, 1865). Conspicuously absent from both discussions is any attempt at a physiological account of precisely that which made nitrous oxide an attractive object of scientific inquiry: *its anaesthetic effects*. Hermann had quite masterfully proposed the physio-chemical mechanism by which N_2_O entered the blood and even sought to demonstrate the sense in which it was actually deadly to inhale, but was want for an account of how nitrous oxide was anything more than a noxious gas.

Protagon would function as just such a synthesizing concept, drawing together Hermann’s disparate observations and bringing them into conversation with existing ideas surrounding the relationship between brain fats and anaesthetic effect. Like many of his peers, Hermann appears to have been ingratiated by Liebreich’s rhetorical sublation of pre-existing investigations into the chemical composition of brain fats, readily taking up the ontological claims of Liebreich’s protagon. Shortly after the publication of Liebreich’s research on protagon, Hermann moved to interpolate the protagon concept into his ongoing research on blood chemistry with the publication “De la présence de la protagone dans le sang.” What is the connection between protagon, anaesthesia, and the blood? Blood is far from an obvious place to seek out a fatty molecule otherwise limited strictly to the domain of brain tissue. But Hermann was also uniquely positioned in this regard. Hermann’s ongoing study of the absorption of gases in the blood prefigured both where and how he would look. Defibrinating blood samples in an excess of warm water and ether, evaporating it at a low temperature, and washing out the cholesterine with cold ether, Hermann obtained pure protagon (Hermann, 1866a). Unsatisfied with the brain for a kingdom, protagon had now been found in the blood (Hermann, 1866a). A non-substance a year prior, the genesis of protagon as a novel bodily entity had almost immediately seen it found all throughout the body.

Now in the blood, Hermann had the basis of a possible connection, murky but nevertheless physiological, between the physio-chemical effects of anaesthetic gases on the blood and changes in the brain. This placed protagon at the crossroads between Hermann’s research on the effect of anaesthetic gases on the blood and the medical phenomena of anaesthesis. “Ueber die Wirkungsweise einer Gruppe von Giften” is the result of just such an alignment of theoretical focuses. Hermann acknowledged the body of literature corroborating the existence of a body of vapour anaesthetics with remarkably similar properties, including chloroform, amyl nitrate, chloroethyl, and other chlorine substitutes, as well as ether, vinegar ether, ethyl-, methyl-, and amyl- alcohols, as did methyl chloride and the olefiant gases (Hermann, 1866b; Perkins-McVey, 2024).^41^ Hermann now hoped to explain the physiological effects of this diverse grouping of substances, most of all by developing a connection between protagon and anaesthetic drugs in the blood.

One of the theories considered by Hermann was that anaesthetic compounds functioned through the dissolution of red blood cells. There were strong intimations of such an effect in his 1864/1864 research on nitrous oxide. At high concentrations in a liquid anaesthetic, red blood cells appeared to dissolve under the microscope, a theory evidently rooted in his earlier observation that NO_2_ altered hemoglobin structure (Hermann, 1866b). Yet, when methylene chloride was bubbled through oxygenated blood, Hermann observed how the presence of methylene chloride broke up rouleaux of red blood cells, and even engendered a discernible swelling in the cells, but the cells themselves failed to disintegrate (Hermann, 1866b, p. 32). Hermann considered whether dissolution was instead a gradual process emergent of the displacement of oxygen or free hemoglobin—yet another theoretical extension on his earlier work on NO_2_. But this model raised an assortment of questions for which Hermann had no answer (Hermann, 1866b). How could dissolution of red blood cells or hemoglobic structures induce anaesthesis when anaesthesia occurred despite variability in the immediate effect on cells (Hermann, 1866b)? Why were anaesthetized subjects not constantly jaundiced (Hermann, 1866b)? Assuming symptoms of dyspnea were allayed by the anaesthetic effects, how could frogs—a creature considerably less susceptible to respiratory impairment—be so readily anaesthetized (Hermann, 1866b)? How come invertebrates with colourless blood can still be anaesthetized (Hermann, 1866b)? Hermann’s blood cell theory was left wanting for answers to any of these questions.

The answer it would seem was protagon, the fatty substance found not merely in the brain and blood but, as Liebreich initially proposed and Hermann later corroborated, wherever we find glycerol-phosphoric acid (Hermann, 1866b).^42^ Using Liebreich’s method, Hermann had already demonstrated that it was trivially easy to acquire protagon from the blood, and that he had personally observed the minute effect of protagon on red blood cells, sufficient—in Hermann’s estimation—to produce anaesthesia (Hermann, 1866b, p. 34, 37). Blood even contained decomposition products of protagon (Hermann, 1866b, p. 36).

The simple, though elegant, solution was that anaesthetizing vapours altered blood chemistry in such a way that protagon was released, where it could then further compound the presence of protagon in the nerves. This theory was supported by the microscopic observation that anaesthetic agents broke up rouleaux of red blood cells and observable changes in individuated cells and the presence of protagon decomposition products in the blood. It was further corroborated through the observation that other rouleaux dissolving substances, such as bile acids and sodium bicarbonate, produced effects similar to acute ether poisoning when injected into the blood stream of a dog (Hermann, 1866b). In these cases, Hermann observed how acute experimental exposure had an intense effect on the central organs, expressed as a slight state of excitation followed shortly by cardiac death (Hermann, 1866b, p. 35). Such effects, it was reasoned, mirrored those of ether and chloroform, which, like ammonia, excite nervous tissue in the period immediately preceding rapid inexcitability and paralysis (Hermann, 1866b, p. 36). What emerges from this line of inquiry is the remarkable suggestion of an analogous relationship between protagon and anaesthetic substances.

Hermann’s arrival at such a striking conclusion typifies “Ueber die Wirkungsweise” as a classic representation of the scientific paper as a rhetorical instrument. From its extensive elucidation of the effects of various anaesthetics on red blood cells to the litany of potent refutations levelled against his own blood dissolution theory, the structure of Hermann’s paper turned on the realization of a conceptual problem, one that needed to be overcome by way of some alternative proposal, even a *deus ex machina*. This is not to suggest Hermann was disingenuous in his exploration of the cell dissolution theory of anaesthetic effect. It was consistent with the methodological and conceptual focuses of Hermann’s broader research program, then centred largely on the physiology of respiration. But it can hardly be ignored that any detailed exposition of the cell dissolution theory only comes into focus via its negation, that the cell dissolution theory as a distinct concept is only formed after the fact, with Hermann having already established the presence of protagon in the blood. Nor can it be ignored that cerebral fats were then still the subject of intense research or that Bibra and Harless had already proposed a fat-solvation hypothesis. This becomes readily apparent in contrasting the forcefulness with which Hermann problematizes the blood dissolution theory with his willingness to mobilize his argument around the novel, and yet considerably more vague, protagon theory. The appeal of protagon theory was rooted in so much more than its ability to ‘better answer questions.’

A possible account for Hermann’s personal affinity for the protagon theory can be attributed to protagon’s presence at the historical crossroads of Darwinism, fat theory, and the grand quest for the physiological basis of mental life. The protagon concept may have levied popular scientific interests in evolutionary theory. Mere utterance of the name ‘protagon’ conjures up then current discussions concerning the simple, single cellular origins of life. Integrating Lorenz Oken’s naturphilosophical theory of infusorial “*Urschleim”* with Darwinian/Neo-Lamarckian thought, Ernst Haeckel’s, 1866*Generelle Morphologie der Organismen* proposed an autogenic origin of life rooted in the existence of an originary protoplasmic mass (Haeckel, 1866; Houe, 2022; Oken, 1805). The single cellular nucleus likewise carried the components involved in hereditary transmission (Haeckel, 1866). A year later in 1867, Thomas Huxley wrote to Haeckel of his “discovery” of *Bathybius haeckelii,* purported to be a sample of a primordial organic agent snatched from the mud bed of the North Atlantic Ocean floor, and named for Haeckel himself (Houe, 2022; Huxley, 1868).

Protagon was the adamic fat, the originary substance from whence the sum of particular cerebral substances were derived. To this point, evolutionary concepts were theoretically significant to Hermann as a hypothetical framework backgrounding the approach to physiology modelled by his Berlin *milieu.* Hermann was, like his mentor du Bois-Reymond, invested in Darwinian theory’s explanatory potential for a strictly physio-chemical science of organized beings (Finkelstein, 2006, 2019).^43^ The *Handbuch der Physiologie,* undoubtedly Hermann’s most popular work and read by both Galton and Darwin, is replete with references to Darwin (Galton, 1879; Hermann, 1881). Nor was Hermann merely an unreflective, happenstance recipient of Darwinist thought. Hermann’s *Handbuch* engages fairly extensively with the issue of autogeny and the origins of life (Hermann, 1881; Peretó & Català, 2012).^44^ Despite going to great length to problematize various different forms of autogeny (particularly that of Haeckel), Hermann ultimately “holds it to be true that autogeny (Urzeugung) must be assumed” (Hermann, 1881, p. 143).^45^

The genesis of the protagon and its ready uptake in 1866 was no accident. The conceptual structure of protagon theory, an adamic fat whose decomposition products participate in the excitation of various bodily processes, echoed those of evolutionary branches peeling away from the first species. To echo the Haeckelian refrain, ontogeny recapitulates phylogeny (Haeckel, 1866). Protagon thus materialized in the midst of wider debates centred on the dynamics of origins and singularities, in spite of divergent schools of thoughts, movements, and disciplines. Hermann would even come to draw an analogous equivalence between chemical reactions and sexual reproduction (Hermann, 1881). That such an originary substance was a cerebral fat was hardly escapable. The nature of brain fats had been a, and perhaps the, defining characteristic of the research program of brain tissue analysis from the late-18th/early-nineteenth century into the 1860s, a decentralized focus of research which had seen the emergence of a staggering array of novel entities of which protagon was the culmination. It had been this elusive search which had conditioned the horizon of possibility in Bibra and Harless’s effort to divine a chemical basis for ether anaesthesia, as well as Hermann’s approach to accounting for anaesthesia as a general category of physiological phenomena. By way of Hermann’s own chemical-evolutionary analogy, protagon was the originating, adamic ‘species’ from which all neural ‘species’ descended.

For Bibra and Harless, the phenomenon of anaesthesia—quite possibly all drug effects—was precipitated by the chemical dissolution of cerebral fats. Ether dissolved fatty materials in the brain and thereby produced its effects. Brain fats were passive substances *vis à vis* the solvation capacity of ether, their quantitative absence being equal to the degree of anaesthesis.^46^ The genesis of protagon as a novel bodily entity fundamentally altered this equation. Hermann’s protagon is not merely a passive substance. It is the overt causative agent in the effect of anaesthesia. Ether, chloroform, amyl nitrate, these substances merely free up an acute concentration of protagon, which instead functions as a form of mediating substance. The emergence of protagon as a novel experimental entity fundamentally altered the nature of the fat theory of anaesthesia, redefining the role of fat in the phenomenon of anaesthesis. With evolution from first species as an analogy, fatty brain matter became propagating agents in the precipitation of powerful physiological effects.

In the years that followed, the protagon concept would find itself under sustained attack, most famously by the English physician Thudichum, though it would cling for dear life up to around 1910 (Sourkes, 1995). As late as 1905, we still find publications seeking to determine whether protagon was a mixture or a compound (Posner & Gies, 1905).^47^ Hermann himself softened on the idea relatively quickly. His 1874 *Lehrbuch der experimentellen Toxicologie* grants protagon nary a mention (Hermann, 1874). It was the propagating function of protagon, divorced from the context of its initial emergence, that lived on in the rapidly developing field of pharmacology. J. Pohl’s, 1891 “Ueber Aufnahme und Vertheilung des Chloroforms im thierischen Organismus” drew heavily on Hermann’s analysis, determining that the solvation of anaesthetic compounds *in,* and not *of,* nervous fats accounted for their effects (Hintzenstern et al., 2002; Pohl, 1891).^48^ When the pharmacologist Hans Horst Meyer postulated the influential observation that anaesthesis occurred in proportion to the substance’s partition coefficient in fat, Meyer upheld the receptive role of cellular fat in the propagation of anaesthetic effect (Lipnick, 1989; Meyer, 1899). Hermann too, much as he was willing to offload the protagon concept, maintained that anaesthetic effect was mediated by fat (lipoids) (Hermann, 1874). Protagon’s short life as a bodily entity, sustained through evolutionary analogy and emergent of broader efforts in organic principle analysis which centred on brain fats, had demonstrably affected the theoretical conception of anaesthetic function.

## Disappearing entities and the role of phenomena

The short and messy story of protagon helps us understand how and why biochemists and pharmacologists turned to the lipoidal theory of anaesthesia. But, more than that, it helps us understand other ‘disappearing acts’ in the historical sciences of life. Protagon, the adamic fat, came with explicit methods of extraction, its own chemical formula, and instrumental use in an experimental setting. It was a tangibly *real* entity. And yet, protagon turned out to be an experimental hallucination, an entity that wasn’t. What are we to make of this?

In 1897, Eduard Buchner noted the presence of carbon dioxide when sugar was introduced to completely ground yeast cells, taken to imply that fermentation was occurring without the assistance of living cells (Bechtel, 2017; Buchner, 1897). Buchner called the singular soluble extra-cellular entities ‘zymase’ (Buchner, 1897; Kohler, 1975). This suggestion was, among other things, an apparent affront to Louis Pasteur’s influential argument that fermentation was vital in nature, a form of airless respiration, and thus set the foundations of modern biochemistry (Barnett, 2000; Kohler, 1975; Pasteur, 1857). But Buchner’s experiment was hardly the protoplasmic theory of life’s *coup de grace,* as it has classically been understood (Bechtel, 2017; Kohler, 1975). Buchner’s research was instead readily taken up by a body of researchers already partial to a biochemical perspective on life, who had already begun to speak of the cell, spatially and temporally, as a kind of factory, outfitted with all manner of biochemical ‘machinery’ (Bechtel, 2017; Kohler, 1975; Reynolds, 2018).^49^

Like protagon, zymase emerged in the midst of lively debates surrounding fermentation and the nature of vital processes, a field of study still bolstered by the programmatic successes of Pasteur. It further turned on the intersection of various fields of science and lines of inquiry, unfolding accidentally from Buchner and his brother's immunological research into the role of proteins (Kohler, 1975). Like protagon, zymase’s discovery as a discrete entity engendered a flurry of research interest, surging across institutional and disciplinary boundaries (Barnett, 2003). And, finally, like protagon, zymase faded out of existence. Standard presentations favour the suggestion that zymase was gradually displaced by a diverse set of different cooperative enzymes (Barnett, 2003). But this disregards the ontological reality of zymase as a discrete entity, however short-lived. It was, until it wasn’t.

In the cases of protagon and zymase, their tangible *thingness* pressed up against the world, moving methods, theories, concepts, entities, and people around them in their ontological forcefulness. They were what Theodore Arabatzis calls ‘hidden entities,’ mind-independent epistemic things which only pass into awareness by way of historically situated methodologies, experimental apparatuses, theoretical frameworks, and partial human encounters (Feest & Sturm, 2011). Zymase and protagon were, in this sense, as *real* as the electron. This has interesting implications for Ian Hacking’s entity realism, typified through the oft-repeated slogan "if you can spray them, then they are real" (Hacking, 1983, p. 24). The tale of protagon might be levied in rebuking this notion, exemplifying not the restrictiveness of Hacking’s position but—after the fashion of Gelfert—the excessive permissiveness of Hacking’s instrumentalism (Gelfert, 2003). The point here, contrary to Gelfert’s critique, is that it *does not matter*.

The permissiveness critique loses sight of the very morass that entity realism seeks to overcome: that experimental *phenomena* mediate the various ways we come to know unseeable entities (Hacking, 1983). Phenomena have a kind of independent existence (Mättig & Stöltzner, 2025). Phenomena and their qualities are what emerge in the process of experimentation, what theories attempt to explain, and it is phenomena that stand at the heart of the instrumentalist criteria typically entailed by entity realism (Mättig & Stöltzner, 2025).^50^ The electron was initially conceived of as a particle, then as a quasi-wave, quasi-particle, and then more fundamentally as a probabilistic wave function, all in accordance with historical variances in phenomena, method, experimental practice, and theory. The kinds of ways that an electron might be regarded as an entity were always pre-arranged through its unique circumstances of its historico-temporal embeddedness. Had what we call ‘electrons’ not first emerged as particles, but something else, it is difficult to say whether it would still be considered a quasi-particle at all. The ‘quasi-particle’ is, in this sense, scaffolded by its historically antecedent phenomena, as well as the concepts and theories that accompanied them. We might go as far as saying the ‘quasi-particle’ *is* its antecedents, as much through their outright influence on its conceptual contents as through their exclusion. In short, the permissiveness of instrumental realism embodies two distinct and yet interrelated dynamics, the epistemic independence of phenomena and the associated indefiniteness of any ontological claims in experimental realism.

How does our protagon story figure in here? Neither protagon nor zymase subvert entity realism. But they do drag entity realism just a little bit further into the "philosophical minefield" of phenomena (Hacking, 1983, p. 122). Protagon was, in the normative sense, *merely* a phenomenon. And yet, as we have seen, even a *mere* phenomenon like protagon can inform the theories, entities, and concepts that gather in association with the later lipoidal theory. Earlier phenomena leave traces, often despite, and sometimes because of, our best efforts to erase them. Experimental phenomena, much like the experiments themselves, have “a life of their own” (Hacking, 1991).^51^

Protagon was a ‘real’ bodily entity with theoretical implications still felt to this day. Modern iterations of the lipoidal theory of anaesthesia uphold the active receptive function of lipids, a concept initially introduced by Hermann’s protagon theory of anaesthesia and carried forward by Meyer-Overton (Overton, 1901; Petersen et al., 2020). Other theories of anaesthetic function has risen in competition, but the lipoidal theory still holds strong as the dominant account of anaesthesis (Petersen et al., 2020). By the time protagon had passed out of being, the traces left by the protagon phenomenon has already participated in constituting the candidacy of an entirely different set of claims for truth or falsehood. The residual influence of phenomena, of protagon, can still be felt. The modern lipoidal theory stands on a historical scaffold built, in no small part, by the protagon theory. Protagon—like zymase—left traces, each forming the conceptual scaffolding for later theories.

## Conclusion

It is not without a tinge of irony that one of the first expressions of opposition to the protagon theory of anaesthesia came from none other than Oscar Liebreich, the father of protagon himself. Couched in a profoundly impactful article outlining the therapeutic significance of chloral hydrate, Liebreich proposed that Hermann’s theory was unfeasible, as protagon could not be found in the blood, and that, while chloral was converted to chloroform in the blood, the physio-chemical processes underlying chloroform’s effects were then impossible to determine (Liebreich, 1869). Protagon, and the associated protagon theory of anaesthesia, had been the subject of rebuke almost from its conception. Yet, it makes for a compelling case study in how even short-lived entities can mobilize theory to great effect.

Historians have largely ignored the story of protagon and Hermann’s protagon theory of anaesthesis. Part of this can be explained by Hermann’s limited fame outside of the nineteenth century, surrounded as he was with the historical giants of German physiology. For Liebreich too, his fame is secured by his discovery of chloral hydrate’s therapeutic effects, rather than his parallel work in organic principle analysis. And yet, Hermann’s work on protagon can be found in the leading journals of the time, cited by those who would go on to establish the modern lipoidal theory of anaesthesia. Therein may lie an explanation for its historiographic obscurity: the protagon theory has hitherto been understand solely as a step toward the lipoidal theory, obscuring its particular distinctiveness and undermining its conceptual contributions.

This has been a brief history of protagon and how its brief streak across the night sky of nineteenth century physiological pharmacology may have had an indelible effect on the subsequent formation of the lipoidal theory of anaesthesia. The emergence of cerebral fat as a central focus of organic principle analysis helped establish fat as an avenue of investigatory focus in the physio-chemical encounter with the new phenomenon of anaesthesia. Conditioned by the then foregrounding investment in brain fat, the protagon concept drew a timely analogy with prominent debates surrounding evolutionary theory and origins of life, culminating in rise and fall of a brief, albeit influential, experimental entity. It was the genesis of protagon as a novel bodily entity which transformed Bibra and Harless’s rudimentary fat-solvation theory into a model premised on cerebral fat’s function as an active mediating substance. Though little studied, protagon has nevertheless fundamentally helped shape the ‘everyday’ medical practice of anaesthesia and warrants further attention.

## Footnotes

1 There was significant ambiguity concerning how the state should be defined. Phrases such as ether or chloroform ‘narcosis’ were frequently used, while it was simultaneously noted that the effect was distinct from those of other known hypnotics or narcotics.

2 Although the language of ‘historical ontology’ is most strongly attached to Ian Hacking where it informs the development of ‘human kinds,’ I take my lead here from Klein and Lefèvre in applying the language of historical ontology to materials and scientific objects (Hacking, 2002; Klein & Lefèvre, 2007). Anaesthesia in particular and medicine in general offers particularly fertile ground upon which to explore the dynamic relationship between these two formulations of ‘historical ontology.’

3 The terminology was generally quite flexible in its use, but “proximate principles,” such as fats, referred to constitutive compounds and “simple principles” generally applied to elements.

4 John proposed that human brains contained a significant phosphoric component which was largely absent in the brains of lower animals, a claim later repeated by Bibra (John, 1814).

5 Gmelin had already set the groundwork for the work published alongside Tiedemann in the years prior (Gmelin & Tiedemann, 1826).

6 “la question de la vie, de l'intelligence”.

7 “[…] futiles, ne conduisant à aucune explication physiologique satisfaisante”.

8 Specifically, Couërbe’s miscroscopic analyses were limited to comparisons in crystalline structure (Couërbe, 1834).

9 This disagreement between Couërbe and Frémy would spark a charged academic exchange between Couërbe over the identity and chemical nature of their respective brain tissue extracts. Liebig credits the initial discovery of Cerebrinsäure to Vauquelin, while simultaneously acknowledging that it was Frémy who did so definitively (Liebig, 1842:2, 109).

10 The historian Ferdinand Gregorovius met Bibra in his wildly decorated home, describing him as "the most eccentric of all Freiherrn that I have yet seen" (Gregorovius, 1907, p. 414). Bibra is in many ways comparable with Humboldt, albeit a generation removed. However unlikely the duo, we might also argue that Bibra’s non-specialist perspective was important in bridging the gap between animal chemistry, medicine, and physiology.

11 Harless compared the intoxicating effects to that of drinking champagne, after which Bibra and Harless offer what might be described as their two distinct phenomenological descriptions of ether’s effects on their respective sense and sensation (Bibra & Harless, 1847).

12 Though they acknowledge that the method was imperfect, Bibra and Harless attached theories of metabolism to urine output and content. That ether reduced the presence of urea in the urine and increased volatile salts was taken to suggest that ether had an effect on the metabolic process (Bibra & Harless, 1847, 164). It was likewise noted that perceived metabolic rates were tied to the duration of ether narcosis, thus explaining the differences in ether’s effects on carnivorous mammals (highly active) and amphibians (less active) (Bibra & Harless, 1847).

13 More specifically, Bibra mixed cerebral fats with sodium bicarbonate and saltpeter, deposited the mixture in a platinum crucible, and burned them together, before dissolving the by-product in a solution of water and hydrochloric acid, finally adding ammonia and precipitating the phosphorus with Epsom salts (Bibra, 1854, p. 98).

14 “die physiologische Bedeutung des Gehirnes von Fett zwar nicht allein abhänge, *aber doch grösstentheils bedingt sei*” [Translation mine. Emphasis added].

15 […] “Fett ein integrirender Bestandtheil des Gehirns” […] “im Gehirne eine ganz andere Rolle zu spielen bestimmt ist, als in den meisten übrigen Organen des Thierleibes”.

16 “Chloroform verhält sich ihm sehr ähnlich und der Gedanke lag eben so nahe, die ganze Reihe alkoholischer Getränke in das Bereich unserer Theorie zu ziehen”.

17 […] “weiß sehr wohl, daß eine Menge anderer Substanzen auf die Nerven einwirken, welche nicht die Fett auflösenden Eigenschaften des Aethers, des Alkohols und der ätherischen Dele besitzen” […] “hiedurch ist aber die Möglichkeit nicht ausgeschlossen, daß diese auf angedeutete Art wirken”.

18 Bibra’s method of measuring changes in metabolism appears to rely primarily on urea extraction (Bibra, 1855).

19 Bibra further remarks at the poor solubility of many opiate alkaloids in water, alcohol, and even ether (Bibra, 1855). By metabolism Bibra here once again draws his conclusion from urine output.

20 “eine ölige, flüssige Masse” It should be noted that Bibra’s awareness of the effects of opium smoking were only second hand.

21 The closest Bibra comes to presenting an alternative theory is a reference to Christoph Hufeland’s Brunonian/Brunonian-adjacent remarks that opium “ist ein Mittel, dessen Kraft in das Innerste, in den Quell des Lebens eingreift,” further adding “daß kaum irgend eine Krankheit eristire, gegen welche, und unter gewissen Bedingungen mit Recht, das Opium nicht angewendet worden sei” (Bibra, 1855, pp. 222–223). Hufeland was famously critical of Brunonianism but later avowed his support for the medical system (Perkins-McVey, 2023).

22 For the sake of brevity, I purposely withhold further discussion of the scope or broader systematic implications of this dialectic, which falls outside the intended scope of this paper. For a discussion of Romantic dialectical selfhood in relation to intoxication see: (Perkins-McVey, 2026).

23 Bibra and Harless were careful to stress that this was not to be understood in reductive terms, and that they would scarcely dare to suggest that the sum of mental life could be defined physio-chemically.

24 “Das Selbst bewusstseyn an sich ist ein Unding; es kann nicht bestehen ohne jene beiden unter einander zusammenhängenden Objecte unserer Vorstellung, deren eines ausser uns, das andere in uns selbst legt”.

25 Bibra and Harless attribute this observation—of the existence of consciousness *sans* self-consciousness—to the repeated experience of etherization itself (Bibra & Harless, p. 83).

26 “physiologisch vermittelt durch die Thätigkeit der Sinnesnerven auf der einen, und die motorischen Nerven auf der anderen Seite, die von dem Centralorgan deren jedesmaliger Stimmung gemäss erregt werden können”.

27 “die Thätigkeit der Sinnesorgane verschwindet,” “keine Willenskraft mehr die motorischen Nerven anregt, und die Seele nur noch in dem Schaffen von affektlosen Vorstellungen und Bildern rastlos beschäftigt ist”.

28 Harless later returned to the nature of self-conscious in his 1861 work of physiological psychology “Der Apparat des Willens” and 1862 book *Die elementaren Funktionen der kreatürlichen Seele* (Harless, 1861, 1862; Pfister & Janczyk, 2012). In both texts, one sees the influence of the theoretical and philosophical questioned raised in his earlier ether experiments with Bibra.

29 “neuen ‘Kategorie’ von Seelenzuständen”.

30 Lösch questions the viability of the fat theory by way of *reductio ad absurdum,* although Lösch acknowledges that chloroform does dissolve fat, possibly faster than sulfuric ether, and that this corresponds with chloroforms faster onset (Lösch, 1848). Lösch suggests that with the evidence at hand the cause of ether or chloroform’s effects can only be speculated upon (Lösch, 1848).

31 The minutiae of the departure from the Lavoisierian embodied in the Liebigian research program of organic chemistry is significant, albeit outside of the purview of this discussion.

32 “die man als Cerebrin, Cerebrinsäure, Lecithin, u. s. w. und als phosphorhaltige Fette bezeichnete, primär nicht im Gehirn existiren.” Emphasis mine.

33 “Ueberall dort, wo von früheren Autoren Glycerinphosphorsäure, Cerebrin u. s. w., überhaupt die sogennanten 'rätselhaften phosphorhaltigen Fette' erwähnt sind, scheint das Protagon im Spiel zu sein, also im Dotter der Eier, Eirer, Sperma, u. s. w. [Hierzu] kommt die von Virchow beobachtete mikroscopische Formenbildung, der er den Namen Myelin beilegt.”

34 “Protagon (von *πρωταγον*)”.

35 Liebreich lets fly a battery of methodological critiques against other brain analyses. Fremy’s isolate is dismissed as a decomposition product on account of Fremy’s use of absolute alcohol on a brain already in the process of natural decomposition (Liebreich, 1865, p. 40). Cerebric acid, separating from alcohol in the form of a barite salt, was thus presumed to be a blend of fatty acids formed by decaying protagon (Liebreich, 1865, p. 40). Similar critiques were made of Wilhelm Müller’s 1858 isolation of cerebrine in Limpricht’s laboratory, which was only extracted from a treated brain (Liebreich, 1865, p. 42). Liebreich’s criticisms might be understood as consistent which the extent to which the methodologies of organic principle analysis, as well as their classification, was still unsettled, and so there was still space for Liebreich to propose a supersessional method.

36 Reference to protagon as the *adamic* fat will be familiar to those acquainted with the concept of adamic language, in reference to a single, original language spoken by humankind prior to the events of the biblical Tower of Babel. With regard to protagon, the adjectival use of “adamic” is better understood in analogy to אדם הראשון [adam ha-rishon](the first man), out of which all of humankind was derived. It is a turn on Liebreich’s description of it as “the mother-substance-of-all,” where Adam Ha-Rishon is the literal and mystical ancestor of all humans, both female and male.

37 Many were convinced of its existence. In 1877, Larmuth and his colleagues recognized that, by Liebreich’s method, a substance matching protagon’s description could always be extracted, while the same could not be said of other proposed proximate principles (Gamgee & Blankenhorn, 1879a, 1879b). Even as figures such as Hoppe-Seyler, Diaconow, and Thudichum attempted to demonstrate that protagon was a mixture of other proximate principles, others rushed to criticize their methods in defense of protagon’s existence (Gamgee & Blankenhorn, 1879a, 1879b).

38 The relationship between Ludimar Hermann and du Bois-Reymond was one of extremes. Initially taken on by du Bois-Reymond only tentatively as an ‘unskilled Jew,’ du Bois-Reymond would write of Hermann as perhaps his best student in 1865, only for the relationship to fall apart in 1867 over dissenting perspectives on the basic nature of electromotor function (Finkelstein, 2006). The bulk of Hermann’s protagon research occurred while the young scientist was still in the good graces of his esteemed mentor.

39 This was quite likely a result of the Davyian assertion, under the influence of Thomas Beddoes, that nitrous oxide was a Brunonian stimulus, and thus in moderate doses engendered a heightening in the vital force (excitability), only to be followed by indirect debility by way of bodily exhaustion and ultimately death (Brown, 1795; Perkins-McVey, 2026). Du Bois-Reymond, alongside Ernst Brücke and Hermann Helmholtz, actively worked to combat the influence of Brunonianism on physiology, a sentiment likewise reflected in the wording of Hermann’s remarks about Davy’s experiments (Perkins-McVey, 2026).

40 “schöne hell carmoisinrothe Farbe.”

41 The existence of a broad class of interrelated anaesthetizing substances, composed primarily of halogenic compounds, had been proposed by Robert Glover as early 1842 and seen significant popularization in the work of Thomas Nunneley (Glover, 1842; Nunneley, 1849; Perkins-McVey, 2024). Glover initially premised his argument for the existence for a novel class of poisons on the basis of the physiological effects of various halogenic compounds, such as chloroform, bromoform, and potassium bromide (Glover, 1842; Nunneley, 1849; Perkins-McVey, 2024).

42 Hermann explicitly singles protagon out as the causative agent: “Das Zusammentreffen der blutkörperchenlösenden und der nervös erregenden und lähmenden Wirkung wäre also nach dieser Ansicht, ein wenn man so sagen darf zufälliges, darin begründet, dass beide Organe einen gemeinsamen Stoff enthalten, auf welchen die Anästhetica gewisse Wirkungen ausüben. Dieser Stoff ist das von O. Liebreich im Gehirn entdeckte Protagon” (Hermann, 1864, p. 33).

43 For du Bois-Reymond, Helmholtz, and others in the Berlin group, Darwin represented a hypothetic framework with which to ground physio-chemical reductionism in the face of widespread vitalism and the latent influence of Romantic science, for example the use of ‘organizing concepts’ in the physiology of their teacher, Johannes Müller (Fullinwider, 1991; Perkins-McVey, 2023). They had done away with vitalism, yet now wanted for an explanatory model. Darwin was acknowledged as being non-experimental and untestable, but a nevertheless compelling explanatory framework.

44 Haeckel’s primordial slime is the most famous argument for autogeny from the nineteenth century, originally a response to the absence of an explanation for the origins of life in *On the Origin of Species* (Peretó & Català, 2012). The case has been made (see: (Strick, 2009)) that Darwin’s initial hesitance was grounded in a desire to appeal to liberal Christian readers. While some, among them Huxley, Haeckel, von Nägeli, Tyndall, and Weissman, looked to prehistory as the site of a primordial abiogenesis, there were some Darwinists, such as Wallace and Bastion, who maintained that autogeny could only be corroborated by a present-day example of spontaneous generation, a position consistent with uniformitarianism (Peretó & Català, 2012). In a letter to Hooker from 1871, Darwin privately argued for primordial autogeny not at the ocean floor, as Haeckel and Huxley proposed, but in a warm pond, where chemical concentrations would conceivably be more acute (Peretó & Català, 2012). By 1881 and likely earlier, Hermann supported the principle of autogeny, proposing an analogy to sexual reproduction implicit to the formation of chemical compounds. An additional parellel might be drawn with Claude Bernard's 1878 protoplasmic theory of anesthesia (Perouansky, 2012). This is all to say that Hermann’s willingness to publicly support an originary, prehistorical autogenesis was far from given or without consequence.

45 “Ich halte für richtig, dass eine Urzeugung angenommen werden muss”.

46 Though murky, the case could be made that this was more than sufficient for Bibra & Harless, insofar as their Romantic conception of *Geist* merely required that the dissolving of brain fats sufficiently disrupt the regular function of the sensory apparatus.

47 This is particularly remarkable owing to the dramatic changes in the study of nervous tissue seen from 1865–1905. This suggests an uneven uptake in protagon as a concept, with some leaving the concept in the nineteenth century while others upheld its existence into the 20th.

48 Hintzenstern et al. (2002) frame Hermann as arguing, much like Bibra and Harless, that anaesthetic vapours function through the dissolution of fat; however, as has already been elucidated, this overlooks the finer details of Hermann’s argument.

49 It was only after Buchner’s publication that Franz Hofmeister fully realized his metaphorical conception of the cellular factory, “with enzymes arranged on colloidal structures like machine tools” (See Reynolds, 2018, p, 65). Reynolds understands the factory metaphor to be essential to the early development of bio-chemistry at the close of the nineteenth century. There are remnants of this framework in science education today: ‘the mitochondria is the powerhouse of the cell.’

50 Phenomena is an incredibly broad category that can be applied as readily to patterns that emerge in the analysis of large data sets as the effects attributed to electrons in a bubble chamber. Here, we use phenomena to denote the tangible example of a proposed organic entity. It is further worth noting the heterogeneity of entity realism. Ronald Giere upholds perspectival realism, arguably a kind of entity realism, wherein he argues for a sharper distinction between the unseen entities employed as research tools (such as protons) and objects of study, with the former deserving stronger realism commitments (Giere, 1988; Khalili, 2024). Citing Giere’s later argument for overlapping perspectives as a methodological strategy for understanding the emergence of realist consensuses, Khalili has recently argued that entity realism and perspectivism is complementary (Giere, 2006; Khalili, 2024).

51 This might be understood as a criticism of entity realism.
